# Hybrid Community–Electronic Health Record Approaches to Apolipoprotein L1 Kidney Disease Screening and Clinical Trials among Black Individuals

**DOI:** 10.1681/ASN.0000001062

**Published:** 2026-03-03

**Authors:** Nadine Barrett, Joab O. Odera, Kenisha Bethea, Maurice Smith, Azita Sadeghpour, Leshon Matthews, Anika Lucas, Ronald L. Godbee, Orlando Dowdy, Leroy Miles, Opeyemi A. Olabisi

**Affiliations:** 1 Division of Population Health Sciences, Department of Social Science and Health Policy, Wake Forest School of Medicine, Winston-Salem, North Carolina; 2 Atrium Health/Wake Forest Comprehensive Cancer Center, Maya Angelo Center for Health Equity, Wake Forest School of Medicine, Wake Forest, North Carolina; 3 Division of Nephrology, Department of Medicine, Duke University School of Medicine, Durham, North Carolina; 4 Duke Molecular Physiology Institute, Duke University School of Medicine, Durham, North Carolina; 5 Duke Aging Center, Duke University School of Medicine, Durham, North Carolina; 6 Duke Clinical and Translational Science Institute, Duke University School of Medicine, Durham, North Carolina; 7 Duke Precision Medicine Program, Department of Medicine, Duke University School of Medicine, Durham, North Carolina; 8 The River Church, Durham, North Carolina; 9 African American Methodist Episcopal Zion Church, Sanford, North Carolina; 10 Enon Tabernacle Baptist Church, Philadelphia, Pennsylvania

**Keywords:** albuminuria, apolipoprotein L1 (APOL1), CKD, clinical trial, community engagement and health, ethnic minority, minority health and disparities, genetic kidney disease

## Abstract

**Key Points:**

- Community engagement achieved high apolipoprotein L1 (APOL1)–mediated kidney disease screening rates but low yield of trial-eligible participants.
- Electronic health record and health care professional referrals identified more participants with *APOL1* high-risk genotype and significant albuminuria.
- Hybrid community–electronic health record recruitment may improve equity and efficiency in APOL1-mediated kidney disease trial enrollment.

**Background:**

Black individuals bear a disproportionate burden of kidney diseases, including genetically mediated risk related to *apolipoprotein L1* (*APOL1*) gene variants. Awareness of APOL1-mediated kidney disease (AMKD) and participation in therapeutic trials remain low. Whether different engagement strategies can raise awareness and identify trial-eligible individuals is uncertain. The Community APOL1 Research Engagement (CARE) study aimed to increase AMKD awareness through culturally tailored education and screening while building a clinical trial–eligible registry, CARE Registry, to support a phase 2 baricitinib trial for AMKD in the Janus kinase-Signal Transducers and Activators of Transcription Inhibition to Reduce APOL1-Associated Kidney Disease.

**Methods:**

The CARE study was conducted from May 2022 to July 2025 across community and clinical settings in multiple US regions, in partnership with churches with predominantly Black attendees. Black adults aged 18–70 years without diabetes or dialysis dependence underwent *APOL1* genotyping and kidney disease screening. Recruitment occurred *via* community events, electronic health record (EHR) queries, physician referrals, and self-referrals. The primary outcome was eligibility for the CARE Registry, defined by *APOL1* high-risk genotype, urine albumin-to-creatinine ratio ≥300 mg/g, and eGFR ≥25 ml/min per 1.73 m^2^.

**Results:**

Of 1052 individuals approached, 789 (75%) consented to screening. Overall, 128 (17%) carried *APOL1* high-risk genotypes. Community events accounted for most enrollments (83%) but yielded low rates of registry-eligible albuminuria (1%). By contrast, EHR queries and physician referrals identified higher proportions of participants with *APOL1* high-risk genotypes and urine albumin-to-creatinine ratio ≥300 mg/g. Twenty-four participants met CARE Registry criteria, and seven enrolled in the Janus kinase-Signal Transducers and Activators of Transcription Inhibition to Reduce APOL1-Associated Kidney Disease trial. Refusal was 4% and attrition was 2%.

**Conclusions:**

Community engagement achieved high participation and awareness but was less efficient for identifying trial-eligible individuals than EHR- and health care professional–based approaches.

**Clinical Trial registry name and registration number:**

ClinicalTrial.gov, NCT05237388.

## Visual Abstract

**Figure:**
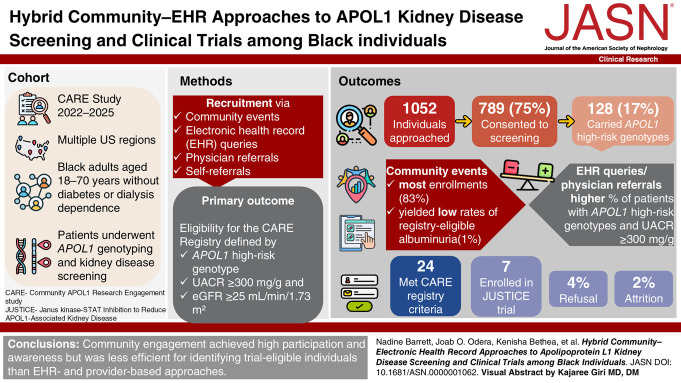

## Introduction

Black individuals bear a disproportionately high burden of kidney failure, developing it at a rate 3.8 times higher than White American individuals.^1^ Although Black individuals comprise only 13% of the US population, they account for 30% of the kidney failure population.^2–4^ A major driver of this disparity are two protein-coding variants of the apolipoprotein L1 (*APOL1*) gene—named G1 and G2—which confer innate protection against *Trypanosoma brucei*, the parasite that causes African sleeping sickness. These *APOL1* variants are found exclusively in individuals with recent West African ancestry.^5–7^ Owing to admixture, these variants are also found in individuals who may not identify as Black but who have recent West African ancestry.^6,8,9^ However, individuals who inherit two risk alleles (G1/G1, G1/G2, or G2/G2) face a markedly increased risk of a spectrum of kidney diseases collectively termed APOL1-mediated kidney disease (AMKD).^5,10,11^ Indeed, although only 13% of Black individuals in the US carry two risk alleles, these individuals account for 50% of Black individuals with hypertension-attributed kidney failure and 70% of those with FSGS.^5,12^

Despite this clear genetic link, AMKD remains largely invisible to individuals and their health care professionals. Among the estimated 6 million Black individuals with *APOL1* high-risk genotypes, 15%–30% are expected to develop AMKD over their lifetime.^9,13^ Routine care rarely includes urine protein screening or *APOL1* testing, even in high-risk families.^14,15^ These gaps, compounded by limited test access and a trust deficit with health care professionals, delay diagnosis and early intervention.^14,16–19^

Recent advances in biomedical research have produced promising therapies targeting the pathobiology of AMKD. The efficacy and safety of these candidates are currently being evaluated in clinical trials.^20,21^ Although Black individuals represent 30% of the US kidney failure population, they account for only 18% of CKD trial participants.^22^ Underrepresentation is often attributed to mistrust linked to historical injustices.^23–25^ However, structural barriers—including health care professional bias, lack of invitations, and limited nephrology access—are equally critical.^26^ Evidence from cancer cohorts shows high willingness to participate when invited and engaged appropriately.^27^ Indeed, the misbelief that Black individuals are unwilling to participate in trials may itself deter health care professionals from extending invitations to eligible Black patients.^26,28,29^

Thus, effective strategies are needed to both raise AMKD awareness and identify those with high-risk genotypes and proteinuria who are trial eligible.^30,31^ Community engagement has shown promise in other fields,^32–34^ but its utility for AMKD is unknown.

To address these gaps, we established the Community APOL1 Research Engagement (CARE) study, a Black-centered initiative designed to raise awareness of AMKD and offer free screening in community settings such as churches and in nephrology clinics. This study also served as a recruitment platform for the Janus kinase/Signal Transducers and Activators of Transcription Inhibition to Reduce APOL1-Associated Kidney Disease (JUSTICE) clinical trial, a randomized, placebo-controlled phase 2 trial testing baricitinib in Black adults with proteinuric CKD and high-risk *APOL1* genotypes, NCT05237388.^20^ Participants with clinically significant kidney disease, indicated by urine albumin-to-creatinine ratio (UACR) ≥300 mg/g, and eGFR ≥25 ml/min per 1.73 m^2^ were enrolled. The trial targets a key APOL1-linked pathway—(Janus kinase-signal transducers and activators of transcription) signaling—which mediates podocyte injury.^35^

## Methods

### Recruitment and Data Collection

The CARE study refers to the overall community- and clinic-based engagement and screening initiative. The CARE Registry refers to the subset of CARE participants who have *APOL1* high-risk genotype, UACR ≥300 mg/g, and eGFR ≥25 ml/min per 1.73 m^2^ who could be screened for enrollment into the JUSTICE trial. eGFR was calculated from serum creatinine using the 2021 CKD Epidemiology Collaboration creatinine equation, which does not include race.^36^ CARE study enrollment reported here was between May 17, 2022, and July 21, 2025, (first consented participant to last consented participant as captured on July 21, 2025). All human participant activities described in the manuscript, including the CARE study and the JUSTICE trial, received prospective review and approval from the Duke University Health System Institutional Review Board before initiation. All participants provided informed consent. The study collected protected health information including basic identifiers such as name, date of birth, phone number, email address, and sex at birth. When a potential participant was not eligible, the Research Electronic Data Capture (REDCap) survey included an option to decline future contact.

Written informed consent was obtained from those who met these criteria and expressed interest in participating. Screening occurred only after consent was signed and included the collection of certain vital signs, when possible (weight and height for body mass index [BMI]), urine dipstick samples, and saliva samples for *APOL1* genotyping. These samples were collected by the study coordinator or a mobile phlebotomy service.

### Variables and Definitions

The primary exposure was recruitment strategy, categorized *a priori* as community events (including faith-based and other community venues), electronic health record (EHR) query, physician referral, or self-referral.

The primary outcome was meeting CARE Registry eligibility criteria, defined as *APOL1* high-risk genotype with albuminuria in the registry-eligible range (UACR ≥300 mg/g) and eGFR ≥25 ml/min per 1.73 m^2^. Secondary outcomes included prevalence of *APOL1* high-risk genotype, prevalence of albuminuria/proteinuria above prespecified thresholds, and subsequent eligibility for and enrollment into the JUSTICE clinical trial among registry-eligible participants.

Prespecified participant characteristics included age, sex assigned at birth, and recruitment geography (state/city). Clinical screening measures included urine dipstick protein category, measured UACR when obtained, and UACR (measured when urine dipstick protein ≥+1). For participants identified through the EHR and clinic workflows, kidney function (eGFR) and urine albumin measure were abstracted from the EHR.

Because recruitment strategy was not randomized, comparisons across recruitment strata were interpreted descriptively. We considered differences in age, sex, and geography as potential confounders of recruitment-yield comparisons. We did not prespecify formal interaction testing; comparisons are descriptive and stratified by recruitment strategy.

### Community Events

Our community engagement strategy was anchored by partnerships with churches with predominantly Black attendees across several states including North Carolina, Pennsylvania, and Virginia. These churches have established long-standing trust and credibility in their communities. To help build trust with the community, the church leadership invited the study team to present accessible information about the high burden of kidney disease in the Black community, the contribution of *APOL1* genetic variants to this burden, the lack of awareness of AMKD, the importance of clinical trials to discovery new therapies, and the perennial underrepresentation of Black individuals in therapeutic trials. The study team also acknowledged history of racism and misdeeds in biomedical research and highlighted steps taken to prevent similar events in this study.

To demonstrate the importance of the study and to help mitigate distrust, many of the church leaders were the first to volunteer to enroll in the study by providing biological samples for screening. This strong endorsement encouraged many congregants to ask questions and subsequently enroll in the study. The church partners are listed in the Acknowledgments. Recruitment also occurred at civic events including Juneteenth celebrations (North Carolina), community health fairs (North Carolina), the Congressional Black Caucus Foundation event (Washington, DC), and class reunion gatherings, among others (North Carolina). Screening and sample collection occurred at these and other venues including a partnership with Duke and a LabCorp-operated Mobile Clinic that facilitated blood and urine collection in remote locations.

### EHR-Based Recruitment

At Duke-affiliated sites, potentially eligible participants were identified through EHR queries using Epic/Maestro care. Queries were designed to identify individuals who self-identified as Black, were between 18 and 70 years of age, and had no documented diagnosis of diabetes, prior solid organ transplantation, dialysis dependence, or recent myocardial infarction or stroke within the preceding 6 months. After health care professional review and approval, identified patients were contacted by the study team, provided with study information, and invited to participate. Individuals who expressed interest completed informed consent remotely or in person and were subsequently provided with home-based sample kits or scheduled for in-person screening visits, as appropriate.

Recruitment was also supported by a study website (https://kidneycareandjustice.com/) that provided study information and facilitated self-referral.

### Physician Referral

Physician referral represented an additional recruitment pathway designed to leverage clinician awareness and engagement. Educational materials originally developed for community outreach were adapted for clinical audiences. The CARE study principal investigator and study team presented this information to clinicians at academic medical centers, outpatient nephrology clinics, and regional and national meetings attended by nephrologists and kidney disease researchers, including the Nephrotic Syndrome Study Network investigators' meeting and conferences organized by NephCure.

Clinicians informed through these venues subsequently identified potentially eligible patients in their practices. After obtaining the patient's permission to share contact information, referring clinicians connected interested individuals with the CARE study team, who then conducted eligibility screening and informed consent procedures independently.

### Self-Referral

A subset of participants entered the study through self-referral. Self-referral occurred through multiple pathways, reflecting both direct and indirect exposure to CARE study activities. Some individuals learned about the study through family members or friends who attended community engagement events. Others accompanied relatives to screening visits and elected to participate after receiving study information. Additional self-referrals arose from exposure to study posters or brochures in nephrology clinics, delayed follow-up after informational sessions, or direct engagement with the study website.

Individuals who self-referred contacted the study team directly and underwent the same eligibility screening, consent, and data collection procedures as participants recruited through other pathways. Participants with positive CKD screening results who had a primary care provider (PCP) received a written report of their urine dipstick screening for protein, UACR (when performed), and eGFR to share with their PCP for follow-up and potential nephrology referral. Those without health insurance or a PCP were referred to nephrology clinics at Duke.

### Urine Testing

Urine dipstick was used as an initial community-screening triage tool because it is inexpensive, rapid, and feasible in nonclinical venues. Participants with dipstick protein ≥1+ underwent confirmatory quantitative UACR testing in a Clinical Laboratory Improvement Amendments–certified laboratory (or contemporaneous clinical UACR was used when available). Dipstick-to-UACR conversion (published approach^37^) was used only for descriptive purposes when measured UACR was unavailable and did not determine CARE Registry eligibility.

### CARE Registry Inclusion and Exclusion Criteria

CARE Registry eligibility required *APOL1* high-risk genotype with albuminuria ≥300 mg/g and eGFR ≥25 ml/min per 1.73 m^2^. Key exclusion criteria included pregnancy, inability to provide consent, and selected conditions that could confound AMKD trial end points (*e.g*., diabetes mellitus, active malignancy, or advanced comorbidity at investigator discretion).

### Data Analysis and Statistical Analysis

Data were analyzed in Python 3.10.9 (NumPy, Pandas, Statsmodels, Scipystats) with *t* tests, pairwise proportion z-tests (Holm correction), chi-squared tests, logistic regression, and ANOVA. Visualizations were developed using Matplotlib/Seaborn; tables were created in Excel. UACR values were derived from labs or estimated from dipstick protein using published formulas.^37^

### Bias, Missing Data, and Loss to Follow-Up

We anticipated selection bias because community-based approaches preferentially reach individuals without a preexisting CKD diagnosis, whereas EHR- and clinic-based approaches may enrich for participants with known or suspected kidney disease. To mitigate differential ascertainment, we used standardized recruitment scripts, uniform consent procedures, and consistent sample handling and laboratory workflows across sites when feasible, with data captured in standardized case report forms and managed centrally in REDCap.

Measurement bias was addressed by prespecifying clinically meaningful thresholds and by retaining measured UACR when available. When measured UACR was unavailable, we estimated UACR from urine dipstick protein using a published conversion approach, enabling consistent categorization of clinically significant albuminuria across recruitment pathways.

Follow-up was defined as completion of the screening workflow (return of biospecimens sufficient for genotyping and urine testing and, when requested, documentation of results disclosure). Loss to follow-up was operationalized as (*1*) loss before sample collection/return (*e.g*., kit provided but specimen not returned after repeated contact attempts) or (*2*) loss after sample collection (specimen obtained but the participant could not be reached for subsequent study procedures and/or results disclosure). Participants with incomplete follow-up contributed available laboratory data where present but were excluded from analyses requiring the missing outcome.

Missing data were handled using variable-specific denominators (complete-case analyses for each outcome). We did not impute missing laboratory or EHR-derived values. The number of participants with missing data for key variables is summarized in Supplemental Table 5.

## Results

### Recruitment Outcomes and Participant Flow

**Figure 1:**
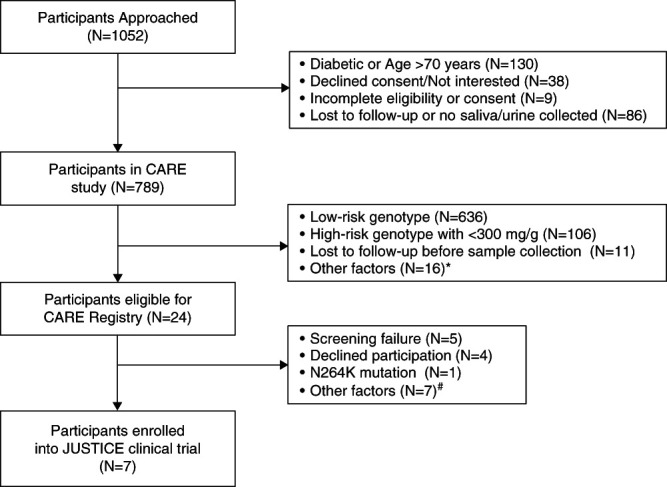
**Overview of recruitment outcomes.** Tree diagram describing the CARE engagement funnel from approached individuals to CARE screening, CARE Registry inclusion, and JUSTICE trial enrollment. *Such as study withdrawal (*n*=4). #Such as lost to follow-up (*n*=3). CARE, Community Apolipoprotein L1 Research Engagement; JUSTICE, Janus kinase-Signal Transducers and Activators of Transcription Inhibition to Reduce Apolipoprotein L1-Associated Kidney Disease.

Over the course of the project, a total of 1052 individuals were approached for enrollment. Of them, 789 individuals (75%) consented to participate in the CARE study (Figure 1). Diabetes or age >70 years were the reasons for half of the exclusions. Refusal (4%, *n*=38) into and the attrition (2%, *n*=18) from CARE study was minimal, challenging long-standing assumptions about Black individuals' unwillingness to participate in clinical research (Figure 1 and Supplemental Figure 1). Ultimately, 24 participants qualified for the CARE Registry. Seven participants from this group were subsequently deemed eligible and consented to participate in the JUSTICE clinical trial. Most participants were from North Carolina (60%) and Pennsylvania (31%), with smaller numbers from Washington, DC, and other states (Supplemental Figure 2 and Supplemental Table 1).

**Figure 2:**
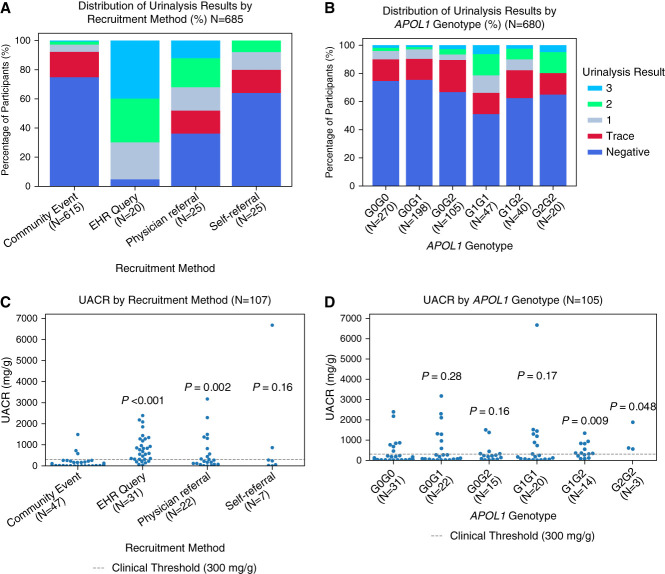
**Distribution of urinalysis.** (A) Recruitment method (*n*=685) and (B) *APOL1* genotype (*n*=680). (A) Stacked bar chart presents data on urinalysis outcomes across various recruitment sources. The *x*-axis categorizes participants by recruitment method, including community event (*N*=615), EHR query (*N*=20), physician referral (*N*=25), and self-referral (*N*=25). The *y*-axis represents the percentage of participants, ranging from 0% to 100%. Each bar is segmented by urinalysis result types: +3 (coral blue), +2 (lime green), +1 (gray), trace (red), negative (navy blue). These segments illustrate the proportion of each result type within each recruitment category, enabling a visual comparison of urinalysis result distributions across different sources of participant recruitment. (B) Stacked bar chart presents data on urinalysis outcomes across various *APOL1* genotypes. The *x-*axis categorizes participants by *APOL1* genotype, including G0/G0 (*n*=270), G0/G1 (*n*=198), G0/G2 (*n*=105), G1/G1 (*n*=47), G1/G2 (*n*=40), and G2/G2 (*N*=20). The *y*-axis represents the percentage of participants, ranging from 0% to 100%. Each bar is segmented by urinalysis result types: +3 (coral blue), +2 (lime green), +1 (gray), trace (red), and negative (navy blue). These segments illustrate the proportion of each result type within each recruitment category, enabling a visual comparison of urine dipstick protein results across various participants' *APOL1* genotype. Five participants had urinalysis results but lacked *APOL1* genotype because of insufficient DNA processed from buccal swabs. Additionally, these five participants were lost to follow-up after sample collection. (C) Swamp plot presents measured UACR across various recruitment methods. The *x*-axis categorizes participants by recruitment method, including community event (*n*=47), EHR query (*n*=31), physician referral (*n*=22), and self-referral (*n*=7). (D) Swamp plot of measured UACR by *APOL1* genotypes. Kruskal–Wallis test was used to test significance in (C) and (D). *APOL1*, *apolipoprotein L1*; EHR, electronic health record; UACR, urine albumin-to-creatinine ratio.

Missing data and follow-up were summarized using variable-specific denominators (Supplemental Table 5). Among the 789 participants who consented to screening, *APOL1* genotyping results were available for 764 (25 missing), and urine dipstick results were available for 685 (104 missing) (Figure 2, A and B). Height and weight were recorded for 289 participants, enabling BMI calculation in this subset. Measured UACR was available for 107 participants (Figure 2, C and D). Loss to follow-up occurred before sample collection (*n*=7) or after sample collection (*n*=31), and 22 participants withdrew or could not be reached.

Most participants recruited to the study were female (63%, Table 1). Thirty-eight declined to consent or were not interested, whereas others were excluded for reasons such as low-risk *APOL1* genotype (*n*=636), high-risk *APOL1* genotype with albuminuria <300 mg/g (*n*=106), or were lost to follow-up (*n*=7). Additional exclusions included stroke, active need of dialysis, and other clinical or logistical factors described as other factors in Figure 1. We conducted follow-ups with all participants by phone, email, or mail. Follow-up was successful in 88% of participants across phone, email, and mail (Table 1).

**Table 1:** Participant flow and key screening characteristics across the Community Apolipoprotein L1 Research Engagement recruitment and enrollment pipeline

Characteristic	*n*/*N*	%
**Screened cohort (consented into CARE, *N*=789)**		
Female sex at birth	497/789	63
Male sex at birth	292/789	37
**Genotyped subset (*n*=764)**		
G0/G0	303/764	40
G0/G1	217/764	28
G0/G2	116/764	15
G1/G2	55/764	7
G1/G1	53/764	7
G2/G2	20/764	3
**Urinalysis subset (dipstick protein, *n*=685)**		
Negative	486/685	71
Trace	115/685	17
+1	42/685	6
+2	26/685	4
+3	16/685	2
**CARE ** **R** **egistry eligibility assessed in screened cohort (*n*=789)**		
CARE Registry—eligible	24/789	3
CARE Registry—ineligible	765/789	97
**Reasons for CARE ** **R** **egistry ineligibility (*n*=765)**		
Low-risk *APOL1* genotype	636/765	83
High-risk *APOL1* genotype with UACR <300 mg/g	106/765	14
Lost to follow-up before sample collection	7/765	1
Other factors^a^	16/765	2
**JUSTICE trial disposition among CARE ** **R** **egistry** **–e** **ligible participants (*n*=24)**		
Enrolled into JUSTICE clinical trial	7/24	29
Not enrolled	17/24	71
*Screening failure*	5/24	21
*Declined participation*	4/24	17
*N264K mutation identified during screening*	1/24	4
*Other factors*^b^	7/24	29

Percentages are calculated within each section denominator. *APOL1*, *apolipoprotein L1*; CARE, Community Apolipoprotein L1 Research Engagement; JUSTICE, Janus kinase-Signal Transducers and Activators of Transcription Inhibition to Reduce Apolipoprotein L1-Associated Kidney Disease; UACR, urine albumin-to-creatinine ratio.

a Other factors (Community Apolipoprotein L1 Research Engagement Registry ineligibility): includes participants who did not meet registry eligibility for reasons other than apolipoprotein L1 genotype and urine albumin-to-creatinine ratio category, such as inability insufficient specimen or unavailable follow-up testing, participant withdrawal after sample collection.

b Other factors (Janus kinase-Signal Transducers and Activators of Transcription Inhibition to Reduce Apolipoprotein L1-Associated Kidney Disease trial non-enrollment): includes participants who did not enroll for reasons other than screening failure, declining participation, or identification of the N264K mutation, such as loss to follow-up after registry eligibility, medical contraindications identified during screening, or logistical barriers to completing trial enrollment.

### Recruitment *via* Faith-Based and Community Venues

**Figure 3:**
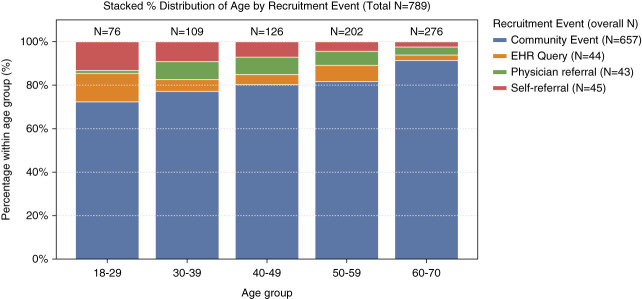
**Distribution of age by recruitment method.** Stacked histogram illustrating the age distribution of participants in the CARE study, stratified by the method through which they were recruited. The *x*-axis represents participant age, while the *y*-axis shows the percentage within each age group. Each bar is divided into colored segments corresponding to specific recruitment methods, allowing for a clear visualization of the contribution of each method to overall recruitment across age groups.

**Figure 4:**
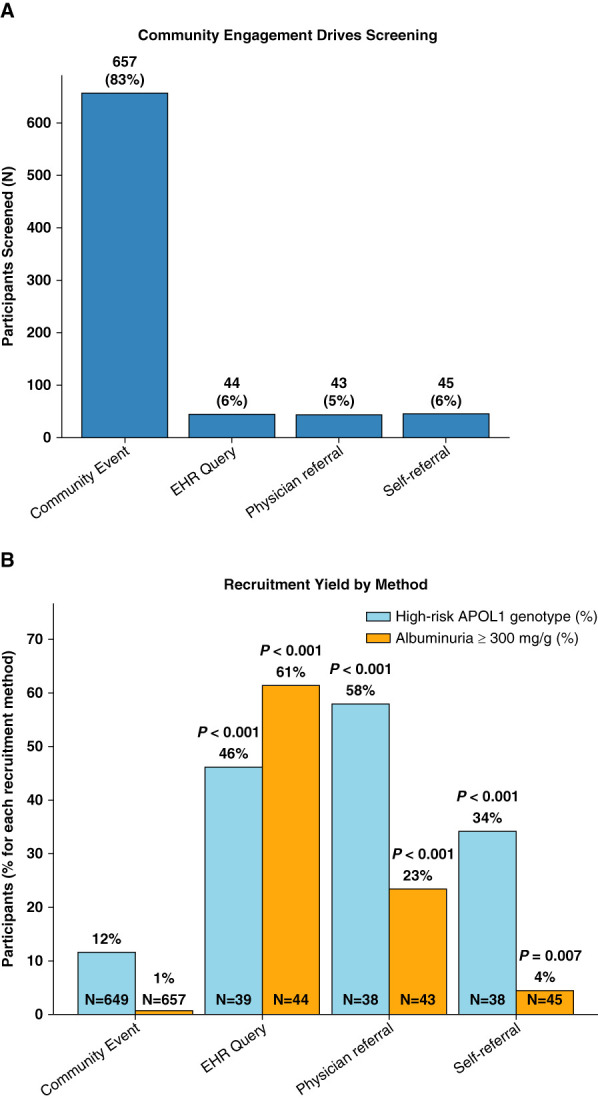
**Recruitment yield by method in the CARE study.** (A) Number and percentage of participants screened by recruitment source. Bars represent the total number of participants enrolled through each method: community events (*n*=657; 83%), EHR query (*n*=44; 6%), physician referral (*n*=43; 5%), and self-referral (*n*=45; 6%). Percentages indicate each method's contribution to the overall screened population. (B) Percentage of participants with high-risk *APOL1* genotype (G1/G1, G1/G2, G2/G2) and albuminuria ≥300 mg/g by recruitment source. Blue bars represent the proportion of genotyped participants with high-risk *APOL1* genotype; orange bars represent the proportion of participants with albuminuria ≥300 mg/g. The total number of participants who had *APOL1* genotyped or had albuminuria measured or calculated from urinalysis (*N*) are shown at the base of each bar. Community event served as the reference group for our Holm adjusted-pairwise z-tests. *P* values indicate statistical significance for pairwise comparisons versus community event using two-sided z-tests for proportions with Holm correction for multiple comparisons.

Community and church engagement recruited most older adults (40–70 years), while younger participants (18–29 years) were more often reached through EHR queries and self-referrals (Figure 3). Community events and local outreach accounted for the vast majority of screenings, achieving high participation with minimal refusal (Figure 4A and Supplemental Table 1). Among 289 participants who had height and weight measurements, majority have BMI above 25, with most having a range between 25 and 40 (Supplemental Figure 3 and Supplemental Table 2).

### *APOL1* Genotype Capture Varies by Recruitment Approach

G0/G0 was the most common genotype, representing 40% of the cohort, followed by G0/G1 at 28%, and G0/G2 at 15% (Table 1). High-risk genotypes were less frequent: G1/G1 and G1/G2 each accounted for 7% and 7%, respectively, while G2G2 represented just 3% of the population. Thus, 17% of participants had *APOL1* high-risk genotype, close to the 13% reported in the literature.^12^

**Figure 5:**
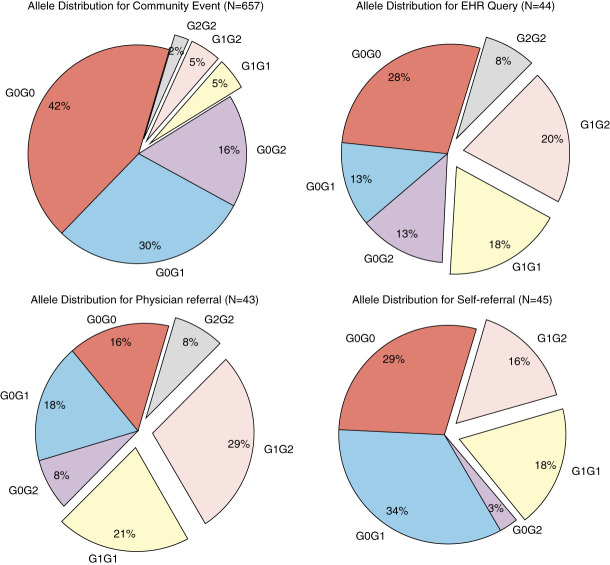
**Allele distribution by recruitment method.** Pie charts depict the distribution of *APOL1* genotypes across different recruitment methods.

Recruitment from community events had a relatively higher frequency of G0/G0 (42%) and low frequencies of high-risk genotypes: G1/G1 (5%), G1/G2 (5%), and G2/G2 (2%) (Figure 5). Compared with participants enrolled in community events, physician referrals, EHR queries, and self-referrals enrolled participants with greater proportions of high-risk genotypes. EHR queries also yielded a substantial representation of G1/G1 (18%), G1/G2 (20%), and G2/G2 (8%), while physician referrals had the highest representation of high-risk genotype, with almost half (58%) of participants recruited by this method having either G1/G1, G1/G2, or G2/G2 genotypes. These genotype distribution patterns (Figure 5) parallel the recruitment yield trends shown in Figure 4B. These results indicate that targeted or self-directed methods may be more effective for identifying at-risk individuals for AMKD clinical trials (Figure 4).

### Lower Albuminuria Observed in Participants Reached through Community Events

As described in the Methods section, urine dipstick testing was used to screen for proteinuria in participants without a known recent history of proteinuria. Participants with ≥1+ dipstick proteinuria underwent confirmatory quantitative UACR measurement. In majority (88%) of participants in community events, dipstick showed no or trace proteinuria (Table 1).

Participants recruited through EHR queries and physician referrals had the highest prevalence of proteinuria on urine dipstick and albuminuria ≥300 mg/g (61% and 23%, respectively) compared with community events (1%) and self-referrals (4%) (Figure 2 and Table 2). Consistent with published reports,^38,39^ high-risk *APOL1* genotypes are significantly associated with an increased prevalence of albuminuria ≥300 mg/g, compared with G0/G0 (Figure 2D). The markedly higher yield of high-risk *APOL1* genotypes and significant albuminuria among participants recruited through EHR queries and physician referrals compared with community events and self-referrals reinforced the conclusion that clinical recruitment is more effective for identifying trial-eligible participants (Figure 4).

**Table 2:** Participant characteristics by recruitment method

Characteristic	Community Event (*n*=657)	EHR Query (*n*=44)	Physician Referral (*n*=43)	Self-Referral (*n*=45)	*P* Value (Overall)
% of total screened	83%	6%	5%	6%	*P* < 0.001^a^
Mean age±SD	53±14	45±15	50±12	43±14	*P* < 0.001^b^
Female sex, *n*/*N* (%)	416/657 (63)	24/44 (55)	27/43 (63)	30/45 (67)	*P* < 0.651^c^
*APOL1* high-risk genotype, *n*/*N* (%)	79/657 (12)	20/44 (46)	25/43 (58)	15/45 (33)	*P* < 0.001^c^
Proteinuria ≥2+ (dipstick), *n*/*N* (%)	20/657 (3)	31/44 (71)	14/43 (33)	4/45 (9)	*P* < 0.001^c^
Albuminuria ≥300 mg/g, *n*/*N* (%)	7/657 (1)	27/44 (61)	10/43 (23)	2/45 (4)	*P* < 0.001^c^

Baseline characteristics of screened individuals stratified by recruitment strategy: community event, electronic health record query, physician referral, and self-referral. Data are shown as mean±SD for continuous variables and as *n*/*N* (%) for categorical variables. Overall *P* values reflect between-group comparisons using one-way ANOVA for age and chi-squared tests for categorical outcomes. *APOL1*, *apolipoprotein L1*; EHR, electronic health record.

a Chi-squared goodness-of-fit test for the distribution of screened individuals across recruitment methods.

b One-way ANOVA computed from group means, SDs, and sample sizes.

c Chi-squared test of independence. See Supplemental Table 4 for *post hoc* pairwise comparisons across recruitment methods. Note that *apolipoprotein L1* genotype was unavailable for eight, five, five, and seven individuals in community event, electronic health record query, physician referral, and self-referral, respectively.

Conversion of urine dipstick protein measurement to UACR, using established formula,^37^ showed a significant and strong correlation between dipstick protein and calculated UACR (Supplemental Figure 4 and Supplemental Table 3), consistent with previous report.^37^ This result justifies our choice of ≥1+ dipstick proteinuria as a liberal threshold for performing UACR testing in potential trial-eligible participants.

Finally, although 789 individuals consented to the CARE study, only 24 participants qualified for the CARE Registry (Table 1). Of these participants, 19 met JUSTICE clinical trial eligibility, and only seven consented to the clinical trial (Tables 1 and 3). Insufficient albuminuria (UACR <300 mg/g) and interval decline in eGFR below 25 ml/min per 1.73 m^2^ were the most common reasons for screening failures for the JUSTICE trial.

**Table 3:** Community Apolipoprotein L1 Research Engagement participants who met Janus kinase-Signal Transducers and Activators of Transcription Inhibition to Reduce Apolipoprotein L1-Associated Kidney Disease clinical trial eligibility by recruitment method

Recruitment Method	Screened (*n*)	Met JUSTICE Eligibility (*n*)	Consented to JUSTICE	NNS
Community events	657	1	0	657
EHR query	44	12	5	3.7
Physician referral	43	5	2	8.6
Self-referral	45	1	0	45
Overall	789	19	7	41.5

Number needed to screen=(number screened *via* recruitment method)/(number meeting JUSTICE eligibility outcome *via* that method). Note that only 19 of the 24 Community Apolipoprotein L1 Research Engagement-registry participants met JUSTICE eligibility. EHR, electronic health record; JUSTICE, Janus kinase-Signal Transducers and Activators of Transcription Inhibition to Reduce Apolipoprotein L1-Associated Kidney Disease; NNS, number needed to screen.

## Discussion

This study addresses a critical barrier to the equitable precision medicine in nephrology: underrepresentation of Black individuals in AMKD trials. Participation rates were higher when recruitment occurred in community settings and through proactive outreach, suggesting that low enrollment in trials may reflect structural barriers in addition to individual-level factors.

Refusal was 4% and attrition was 2%. Consistent with previous reports, participation may be higher when individuals are invited and engaged through culturally tailored approaches.^23–27,40^ Importantly, our results distinguish between reach and yield. Although community-based strategies maximized awareness, consent, and trust, they yielded few individuals meeting stringent genetic and phenotypic eligibility criteria for AMKD therapeutic trials.

By contrast, EHR-based recruitment and physician referrals were markedly more efficient for identifying participants with *APOL1* high-risk genotypes and clinically significant albuminuria. Formal statistical comparisons confirmed significantly higher yields of trial-relevant phenotypes through these clinically anchored strategies. These complementary strengths suggest that no single recruitment modality is sufficient when the goals of equity, efficiency, and scientific rigor must be met simultaneously.

These results have immediate implications for nephrology research design. The contrasting yield profiles suggest that a hybrid model—leveraging community partnerships for population-level engagement and clinical data-driven approaches for targeted recruitment—can simultaneously promote equity and efficiency. Such a model aligns with National Institutes of Health and US Food and Drug Administration diversity enrollment targets and could be adapted across nephrology research networks, particularly for genetically defined or biomarker-driven studies.

A central strength of the CARE Registry was its precision focus on Black individuals with *APOL1* high-risk genotypes and proteinuria, ensuring scientific relevance for AMKD trials. This approach reflects the broader shift toward personalized medicine in nephrology. Consistent with our results section, clinical recruitment methods (EHR queries and physician referrals) enriched for high-risk genotypes and significant albuminuria, yielding more trial-eligible participants.

As in other fields, community components maximize equity and trust but are yield-sparse for stringent phenotypes; clinic/EHR components invert that trade-off—so budgets, staffing, and timelines should explicitly plan for a hybrid funnel rather than expecting one method to do both.^41,42^

Community engagement remains critical, as illustrated during coronavirus disease 2019 when limited outreach contributed to vaccine hesitancy among Black individuals.^43,44^ Similar hybrid models have succeeded elsewhere: the Black barbershop trial in cardiology reduced hypertension,^45^ the project Creating a Higher Understanding of Cancer Research and Community Health built a large Black cancer prevention cohort,^46^ and The Accountability for Cancer Care through Undoing Racism and Equity (ACCURE) study combined EHR registries with community partnerships to reduce disparities in cancer care.^42,47^

Conversely, EHR-enabled strategies excel at efficiently surfacing trial-eligible patients who already meet computable phenotype criteria. The National Patient-Centered Clinical Research Network Aspirin Dosing: A Patient Table Assessing Benefits and Long-Term Effectiveness trial enrolled >15,000 participants *via* health system EHRs.^42^ In oncology, ACCURE pragmatic trial paired real-time EHR registry to community partnership and nurse navigation, improving treatment completion for breast and lung cancer and narrowing Black–White disparities—an instructive hybrid model that coupled data infrastructure to trusted relationships.^48^ Nephrology has long used community screening to expand reach—*e.g*., the National Kidney Foundation Kidney Early Evaluation Program. However, these efforts mainly increased awareness and initial detection rather than yield larger numbers of immediately trial-eligible patients.^41^

Against this backdrop, CARE's result fit a consistent pattern: Community venues maximize reach, consent rates, and trust, whereas EHR queries and health care professional referrals maximize the yield of participants with both *APOL1* high-risk genotypes and clinically significant albuminuria. Similar reach-versus-yield trade-offs are visible when comparing church-based cancer screening and lay-advisor program to EHR alert/navigator oncology models.^49^ CARE therefore operationalizes a pragmatic two-stage funnel: (*1*) community-embedded engagement and screening to build awareness, reduce mistrust, and identify at-risk individuals and then (*2*) EHR/health care professional–driven targeting to enrich for trial-eligible phenotypes. Parallels in cardiology and oncology reinforce that hybrid designs can achieve both equity (who gets invited) and efficiency (who qualifies).^27,45,48^

Future AMKD trials could formalize a two-stage hybrid pipeline: (*1*) deploy community events for wide-net screening and *APOL1* education with immediate urine testing and optional genotyping; (*2*) run monthly EHR sweeps using computable phenotypes to generate health care professional–vetted referral lists; (*3*) add navigation (nurse/clinical research coordinator) to schedule confirmatory laboratories, close eligibility gaps (*e.g*., repeat UACR), and address structural barriers—mirroring ACCURE's alert plus-navigation architecture and barbershop-style “trusted-venue to clinician” linkage.

From an ethical and community-engaged research perspective, it is notable that participants with *APOL1* high-risk genotypes who did not meet registry criteria were not simply screened and dismissed. These individuals received post-test counseling, educational materials, and recommendations for longitudinal kidney health monitoring, reinforcing trust and reciprocity—cornerstones of sustainable community partnership.

This study has several limitations. First, the number of participants ultimately eligible for the CARE Registry and the JUSTICE trial was small, reflecting the stringent eligibility criteria rather than limited engagement. Second, the cohort was predominantly female, potentially limiting generalizability. In addition, the middle age of the enrolled participants may limit generalizability to younger population. Third, community recruitment was largely conducted through faith-based venues in the southeastern United States, which may not capture the full diversity of Black communities nationwide. Fourth, the reliance on dipstick testing as an initial screening tool may have introduced misclassification. However, this approach was selected to enable scalable screening in nonclinical settings, with confirmatory UACR testing performed when indicated.

Finally, while we propose a hybrid recruitment model, this study does not establish causal links between community engagement and subsequent clinical recruitment efficiency. Future studies incorporating participant surveys and longitudinal tracking will be important to evaluate these relationships. Beyond trial enrollment, it is conceivable that in the future, once effective treatments of AMKD are available, community screening might be a suitable tool for identifying at-risk individuals with high-risk *APOL1* genotypes for preventive treatments.

In conclusion, the CARE initiative demonstrates that Black communities are highly willing to engage in APOL1-related research when approached through trusted, culturally responsive strategies. By clearly delineating the complementary roles of community engagement and clinically targeted recruitment, this work provides a practical blueprint for advancing equitable precision medicine in nephrology.

## Supplementary Material

## Data Availability

All original data, including deidentified patient-level data or individual laboratory data measurements, are included in the manuscript and/or supplemental material. Original data generated for the study will be made available upon reasonable request to the corresponding author. Data Type: Statistical Analysis Plan. Reason for Restricted Access: Deidentified individual participant data (including data dictionaries) from the CARE Study will be shared under controlled access, including key demographic, recruitment, *APOL1* genotype category, urinalysis/UACR, eGFR, and recruitment/retention variables used in the analyses. The protocol, REDCap instruments/eCRFs, and analytic code/statistical analysis plan will also be available. Data will be available 3 months after publication for 5 years. Access will be granted to qualified researchers with a methodologically sound proposal and Institutional Review Board approval/exemption as appropriate, following execution of a data use agreement; requests will be reviewed by CARE study leadership and fulfilled *via* secure transfer or a controlled-access repository.

## Declarative Statements

This study includes clinical experimentation and received Institutional Review Board or Ethics Committee approval. All patients provided written informed consent. This study includes clinical experimentation and complies with the Declaration of Helsinki.

## Supplemental Material

Supplemental Table 1. CARE participant recruitment by state and recruitment event.

Supplemental Table 2. Descriptive statistics for key demographic and clinical variables.

Supplemental Table 3. Median UACR and interquartile range by urinalysis category and pairwise *t* test for UACR.

Supplemental Figure 1. Study attrition among CARE participants.

Supplemental Figure 2. Distribution of CARE participants by city and state.

Supplemental Figure 3. Height in inches and BMI distribution of 289 participants who were willing to have both their height and weight measured.

Supplemental Figure 4. Calculated albumin/creatinine ratio (UACR, mg/g) versus dipstick urinalysis.

Supplemental Table 4. Pairwise *post hoc* comparisons among recruitment methods for age, APOL1 high-risk genotype, proteinuria ≥2+ by urine dipstick, and albuminuria >300 mg/g.

Supplemental Table 5. Missingness summary.
