# Supplementary material for: Hybrid Community–Electronic Health Record Approaches to Apolipoprotein L1 Kidney Disease Screening and Clinical Trials among Black Individuals
Source: J Am Soc Nephrol. 2026 Mar 3;37(8):1720–32. doi: 10.1681/ASN.0000001062 (PMC13406261; doi:10.1681/ASN.0000001062)
Supplement: Supplementary file 1 [file jasn-37-1720-s001.pdf]

ASN
American Society of Nephrology
JASN
Journal of the American Society of Nephrology
KIDNEY360
Accessing Our World From Every Angle
CJASN
Clinical Journal of the American Society of Nephrology

ASN Journal Disclosure Form

As per ASN journal policy, I have disclosed any financial relationships or commitments I have held in the past 36 months as included below. I have listed my Current Employer below to indicate there is a relationship requiring disclosure. If no relationship exists, my Current Employer is not listed.

N. Barrett reports the following:
Patents or Royalties: Training program called JUst ASK, copyrighted by Duke and Nadine Barrett, 2019.; and
Advisory or Leadership Role: President and Past President , Association of Cancer Care Centers.

I understand that the information above will be published within the journal article, if accepted, and that failure to comply and/or to accurately and completely report the potential financial conflicts of interest could lead to the following: 1) Prior to publication, article rejection, or 2) Post-publication, sanctions ranging from, but not limited to, issuing a correction, reporting the inaccurate information to the authors' institution, banning authors from submitting work to ASN journals for varying lengths of time, and/or retraction of the published work.

Name: Nadine Barrett
Manuscript ID: JASN-2025-001306R1
Manuscript Title: Hybrid Community-EHR Approaches to APOL1 Kidney Disease Screening and Clinical Trials among African Americans
Date of Completion: January 5, 2026
Disclosure Updated Date: January 5, 2026

ASN American Society of Nephrology
JASN Journal of the American Society of Nephrology
KIDNEY360 Accessing Our World From Every Angle
CJASN Clinical Journal of the American Society of Nephrology

ASN Journal Disclosure Form

As per ASN journal policy, I have disclosed any financial relationships or commitments I have held in the past 36 months as included below. I have listed my Current Employer below to indicate there is a relationship requiring disclosure. If no relationship exists, my Current Employer is not listed.

K. Bethea has nothing to disclose.

I understand that the information above will be published within the journal article, if accepted, and that failure to comply and/or to accurately and completely report the potential financial conflicts of interest could lead to the following: 1) Prior to publication, article rejection, or 2) Post-publication, sanctions ranging from, but not limited to, issuing a correction, reporting the inaccurate information to the authors' institution, banning authors from submitting work to ASN journals for varying lengths of time, and/or retraction of the published work.

Name: Kenisha Bethea
Manuscript ID: JASN-2025-001306R1
Manuscript Title: Hybrid Community-EHR Approaches to APOL1 Kidney Disease Screening and Clinical Trials among African Americans
Date of Completion: January 5, 2026
Disclosure Updated Date: January 5, 2026

ASN American Society of Nephrology
JASN Journal of the American Society of Nephrology
KIDNEY360 Accessing Our World From Every Angle
CJASN Clinical Journal of the American Society of Nephrology

ASN Journal Disclosure Form

Date: 12 Feb 2026

Author: Dr. Orlando Dowdy / od137@mac.com

Manuscript ID: JASN-2025-001306R2

Manuscript Title: "Hybrid Community-EHR Approaches to APOL1 Kidney Disease Screening and Clinical Trials among African Americans."

Disclosure Statements

I have nothing to disclose

ASN American Society of Nephrology
JASN Journal of the American Society of Nephrology
KIDNEY360 Accessing Our World From Every Angle
CJASN Clinical Journal of the American Society of Nephrology

ASN Journal Disclosure Form

Date: 2/12/2026

Author: Ronald L. Godbee

Manuscript ID: JASN-2025-001306R2

Manuscript Title: Hybrid Community-EHR Approaches to APOL1 Kidney Disease Screening and Clinical Trials among African Americans.

Disclosure Statement

I have nothing to disclose

ASN American Society of Nephrology
JASN Journal of the American Society of Nephrology
KIDNEY360 Accessing Our World From Every Angle
CJASN Clinical Journal of the American Society of Nephrology

ASN Journal Disclosure Form

As per ASN journal policy, I have disclosed any financial relationships or commitments I have held in the past 36 months as included below. I have listed my Current Employer below to indicate there is a relationship requiring disclosure. If no relationship exists, my Current Employer is not listed.

A. Lucas has nothing to disclose.

I understand that the information above will be published within the journal article, if accepted, and that failure to comply and/or to accurately and completely report the potential financial conflicts of interest could lead to the following: 1) Prior to publication, article rejection, or 2) Post-publication, sanctions ranging from, but not limited to, issuing a correction, reporting the inaccurate information to the authors' institution, banning authors from submitting work to ASN journals for varying lengths of time, and/or retraction of the published work.

Name: Anika Lucas
Manuscript ID: JASN-2025-001306R1
Manuscript Title: Hybrid Community-EHR Approaches to APOL1 Kidney Disease Screening and Clinical Trials among African Americans
Date of Completion: January 5, 2026
Disclosure Updated Date: January 5, 2026

ASN
American Society of Nephrology
JASN
Journal of the American Society of Nephrology
KIDNEY360
Accessing Our World From Every Angle
CJASN
Clinical Journal of the American Society of Nephrology

ASN Journal Disclosure Form

As per ASN journal policy, I have disclosed any financial relationships or commitments I have held in the past 36 months as included below. I have listed my Current Employer below to indicate there is a relationship requiring disclosure. If no relationship exists, my Current Employer is not listed.

L. Matthews has nothing to disclose.

I understand that the information above will be published within the journal article, if accepted, and that failure to comply and/or to accurately and completely report the potential financial conflicts of interest could lead to the following: 1) Prior to publication, article rejection, or 2) Post-publication, sanctions ranging from, but not limited to, issuing a correction, reporting the inaccurate information to the authors' institution, banning authors from submitting work to ASN journals for varying lengths of time, and/or retraction of the published work.

Name: Leshon Matthews
Manuscript ID: JASN-2025-001306R1
Manuscript Title: Hybrid Community-EHR Approaches to APOL1 Kidney Disease Screening and Clinical Trials among African Americans
Date of Completion: January 7, 2026
Disclosure Updated Date: January 7, 2026

ASN
American Society of Nephrology
JASN
Journal of the American Society of Nephrology
KIDNEY360
Accessing Our World From Every Angle
CJASN
Clinical Journal of the American Society of Nephrology

ASN Journal Disclosure Form

As per ASN journal policy, I have disclosed any financial relationships or commitments I have held in the past 36 months as included below. I have listed my Current Employer below to indicate there is a relationship requiring disclosure. If no relationship exists, my Current Employer is not listed.

L. Miles reports the following:
Consultancy: Maze Theraputics

I understand that the information above will be published within the journal article, if accepted, and that failure to comply and/or to accurately and completely report the potential financial conflicts of interest could lead to the following: 1) Prior to publication, article rejection, or 2) Post-publication, sanctions ranging from, but not limited to, issuing a correction, reporting the inaccurate information to the authors' institution, banning authors from submitting work to ASN journals for varying lengths of time, and/or retraction of the published work.

Name: Leroy Miles
Manuscript ID: 2025-001306R1
Manuscript Title: Hybrid Community-EHR Approaches to APOL1
Date of Completion: January 14, 2026
Disclosure Updated Date: January 14, 2026

ASN
American Society of Nephrology
JASN
Journal of the American Society of Nephrology
KIDNEY360
Accessing Our World From Every Angle
CJASN
Clinical Journal of the American Society of Nephrology

ASN Journal Disclosure Form

As per ASN journal policy, I have disclosed any financial relationships or commitments I have held in the past 36 months as included below. I have listed my Current Employer below to indicate there is a relationship requiring disclosure. If no relationship exists, my Current Employer is not listed.

J. Odera reports the following:
Employer: Eli Lilly - Spouse; and Ownership Interest: Eli Lilly - Spouse.

I understand that the information above will be published within the journal article, if accepted, and that failure to comply and/or to accurately and completely report the potential financial conflicts of interest could lead to the following: 1) Prior to publication, article rejection, or 2) Post-publication, sanctions ranging from, but not limited to, issuing a correction, reporting the inaccurate information to the authors' institution, banning authors from submitting work to ASN journals for varying lengths of time, and/or retraction of the published work.

Name: Joab O Odera
Manuscript ID: JASN-2025-001306R1
Manuscript Title: Hybrid Community-EHR Approaches to APOL1 Kidney Disease Screening and Clinical Trials among African Americans
Date of Completion: January 8, 2026
Disclosure Updated Date: January 7, 2026

ASN American Society of Nephrology
JASN Journal of the American Society of Nephrology
KIDNEY360 Accessing Our World From Every Angle
CJASN Clinical Journal of the American Society of Nephrology

ASN Journal Disclosure Form

As per ASN journal policy, I have disclosed any financial relationships or commitments I have held in the past 36 months as included below. I have listed my Current Employer below to indicate there is a relationship requiring disclosure. If no relationship exists, my Current Employer is not listed.

O. Olabisi reports the following:
Employer: Duke Health/Duke University; Consultancy: Icagen; Maze Therapeutics; Guidepoint; Research Funding: Icagen; Honoraria: Chinook Therapeutics; Guidepoint; and Advisory or Leadership Role: Maze Therapeutics.

I understand that the information above will be published within the journal article, if accepted, and that failure to comply and/or to accurately and completely report the potential financial conflicts of interest could lead to the following: 1) Prior to publication, article rejection, or 2) Post-publication, sanctions ranging from, but not limited to, issuing a correction, reporting the inaccurate information to the authors' institution, banning authors from submitting work to ASN journals for varying lengths of time, and/or retraction of the published work.

Name: Opeyemi A. Olabisi
Manuscript ID: JASN-2025-001306R1.
Manuscript Title: Hybrid Community-EHR Approaches to APOL1 Kidney Disease Screening and Clinical Trials among African Americans
Date of Completion: January 5, 2026
Disclosure Updated Date: January 5, 2026

ASN American Society of Nephrology
JASN Journal of the American Society of Nephrology
KIDNEY360 Accessing Our World From Every Angle
CJASN Clinical Journal of the American Society of Nephrology

ASN Journal Disclosure Form

As per ASN journal policy, I have disclosed any financial relationships or commitments I have held in the past 36 months as included below. I have listed my Current Employer below to indicate there is a relationship requiring disclosure. If no relationship exists, my Current Employer is not listed.

A. Sadeghpour reports the following:
Employer: Duke University Health System; and Ownership Interest: Biogen; GE.

I understand that the information above will be published within the journal article, if accepted, and that failure to comply and/or to accurately and completely report the potential financial conflicts of interest could lead to the following: 1) Prior to publication, article rejection, or 2) Post-publication, sanctions ranging from, but not limited to, issuing a correction, reporting the inaccurate information to the authors' institution, banning authors from submitting work to ASN journals for varying lengths of time, and/or retraction of the published work.

Name: Azita Sadeghpour
Manuscript ID: JASN-2025-001306R1
Manuscript Title: Hybrid Community-EHR Approaches to APOL1 Kidney Disease Screening and Clinical Trials among African Americans
Date of Completion: January 5, 2026
Disclosure Updated Date: January 5, 2026

ASN American Society of Nephrology | JASN Journal of the American Society of Nephrology | KIDNEY360 Accessing Our World From Every Angle | CJASN Clinical Journal of the American Society of Nephrology

ASN Journal Disclosure Form

As per ASN journal policy, I have disclosed any financial relationships or commitments I have held in the past 36 months as included below. I have listed my Current Employer below to indicate there is a relationship requiring disclosure. If no relationship exists, my Current Employer is not listed.

M. Smith reports the following:
Employer: Duke

I understand that the information above will be published within the journal article, if accepted, and that failure to comply and/or to accurately and completely report the potential financial conflicts of interest could lead to the following: 1) Prior to publication, article rejection, or 2) Post-publication, sanctions ranging from, but not limited to, issuing a correction, reporting the inaccurate information to the authors' institution, banning authors from submitting work to ASN journals for varying lengths of time, and/or retraction of the published work.

Name: Maurice Walter Smith
Manuscript ID: JASN-2025-001306R1
Manuscript Title: Hybrid Community-EHR Approaches to APOL1 Kidney Disease Screening and Clinical Trials among African Americans
Date of Completion: January 5, 2026
Disclosure Updated Date: January 5, 2026
