# Supplementary material for: Hybrid Community–Electronic Health Record Approaches to Apolipoprotein L1 Kidney Disease Screening and Clinical Trials among Black Individuals
Source: J Am Soc Nephrol. 2026 Mar 3;37(8):1720–32. doi: 10.1681/ASN.0000001062 (PMC13406261; doi:10.1681/ASN.0000001062)
Supplement: Supplementary file 2 [file jasn-37-1720-s002.pdf]

Supplemental Material

Hybrid Community–Electronic Health Record Approaches to Apolipoprotein L1 Kidney Disease Screening and Clinical Trials among Black Individuals

Nadine Barrett[1,2], Joab O. Odera[3,4,5], Kenisha Bethea[6], Maurice Smith [3,4], Azita Sadeghpour[7], Leshon Matthews[3,4], Anika Lucas [1], Ronald L. Godbee[8], Orlando Dowdy[9], Leroy Miles[10], Opeyemi A. Olabisi[3,4], and CARE Community Partners[11]

[1]Department of Social Science and Health Policy, Division of Population Health Sciences, Wake Forest School of Medicine, Winston-Salem, North Carolina, USA.

[2]Atrium Health/Wake Forest Comprehensive Cancer Center and Maya Angelo Center for Health Equity, Wake Forest School of Medicine, Wake Forest, North Carolina, USA. [3]Division of Nephrology, Department of Medicine, Duke University School of Medicine, Durham, North Carolina, USA.

[4]Duke Molecular Physiology Institute, Duke University School of Medicine, Durham, North Carolina, USA.

[5]Duke Aging Center, Duke University School of Medicine, Durham, North Carolina, USA.

[6]Duke Clinical and Translational Science Institute, Duke University School of Medicine, Durham, North Carolina, USA.

[7]Duke Precision Medicine Program, Department of Medicine, Duke University School of Medicine, Durham, North Carolina, USA

[8]The River Church, Duram, North Carolina, USA.

[9]African American Methodist Episcopal Zion Church, Sanford District, North Carolina USA

[10]Enon Tabernacle Baptist Church, Philadelphia, Pennsylvania, USA.

[11]CARE Community Partners are listed in the Appendix

Table of contents for the Supplemental Material

Supplemental Table 1: CARE Participant Recruitment by State and Recruitment event

Supplemental Table 2: Descriptive statistics for key demographic and clinical variables

Supplemental Table 3: Median UACR and IQR by Urinalysis Category and Pairwise T-test for UACR

Supplemental Figure 1: Study attrition among CARE participants

Supplemental Figure 2: Distribution of CARE Participants by City and State

Supplemental Figure 3: Height in inches and BMI distribution of 289 participants who were willing to have both their height and weight measured

Supplemental Figure 4: Calculated albumin/creatinine ratio (UACR, mg/g) vs Dipstick Urinalysis

Supplemental Table 4: Pairwise post-hoc comparisons among recruitment methods for age, APOL1 high-risk genotype, proteinuria ≥ 2+ by urine dipstick, and albuminuria>300mg/g

Supplemental Table 5: Missingness Summary

CARE Participants (N=789)	**Number of Participants**	**Percentage**
**State/District (N = 789)**		
North Carolina	471	60
Pennsylvania	248	31
District of Columbia	34	4
Other	36	5
**Recruitment Event**		
Community Event	657	83
EHR Query	44	6
Self-referral	45	6
Physician referral	43	5

Supplemental Table 1: CARE Participant Recruitment by State and Recruitment event. Table provides a summary of participant recruitment by both geographic location and recruitment method.

	Median (IQR)	Mean ± SD	Range	Care Participant (%)
Age at consent (N=789)	54.0 (22.4)	51.4 ± 13.9	18.2 - 70.9	100.0
Height (in) (N=289)	65.6 (4.8)	66.0 ± 3.8	56.7 - 77.6	36.6
Weight (lb) (N=289)	191.3 (70.7)	198.1 ± 52.6	69.1 - 413.9	36.6
BMI (N=289)	31.2 (9.0)	31.9 ± 7.8	9.4 - 61.1	36.6
Adjusted UACR (N=789)	8.3 (7.5)	80.1 ± 361.1	2.6 - 6684.0	100.0
UPCR (N=22)	905.0 (1562.0)	1648.3 ± 2425.6	68.0 - 11548.0	2.8
eGFR (N=62)	55.5 (41.0)	60.2 ± 29.3	22.0 - 136.0	7.9

Supplemental Table 2: Descriptive statistics for key demographic and clinical variables. Values are based on non-missing observations. Columns are presented as: Variable (sample size), Median (IQR), Mean ± SD, and Range (minimum–maximum). Variables include: age at consent, height (in), weight (lb), BMI, adjusted albumin/creatinine ratio (UACR), UPCR protein in urine, and eGFR (measures kidney function)

Dipstick Urinalysis Category	Median UACR	UACR IQR		T-statistic	p-value
Negative	8.3	7.7 - 10.7	vs Trace	-26.4	<0.0001
			vs +1	-15.1	<0.0001
			vs +2	-24.3	<0.0001
			vs +3	-32.4	<0.0001
Trace	17.2	15.9 – 24.1	vs +1	-6.5	<0.0001
			vs +2	-11.3	<0.0001
			vs +3	-15.5	<0.0001
+1	62.7	29.3 – 131.8	vs +2	-3.5	<0.0001
			vs +3	-8.3	<0.0001
+2	234	92.3 - 356.9	vs +3	-5.3	<0.0001
+3	1326.5	649.9 - 1925.3			

Supplemental Table 3: Median UACR and IQR by Urinalysis Category and Pairwise T-tests for UACR. UACR is measured mg/g protein.

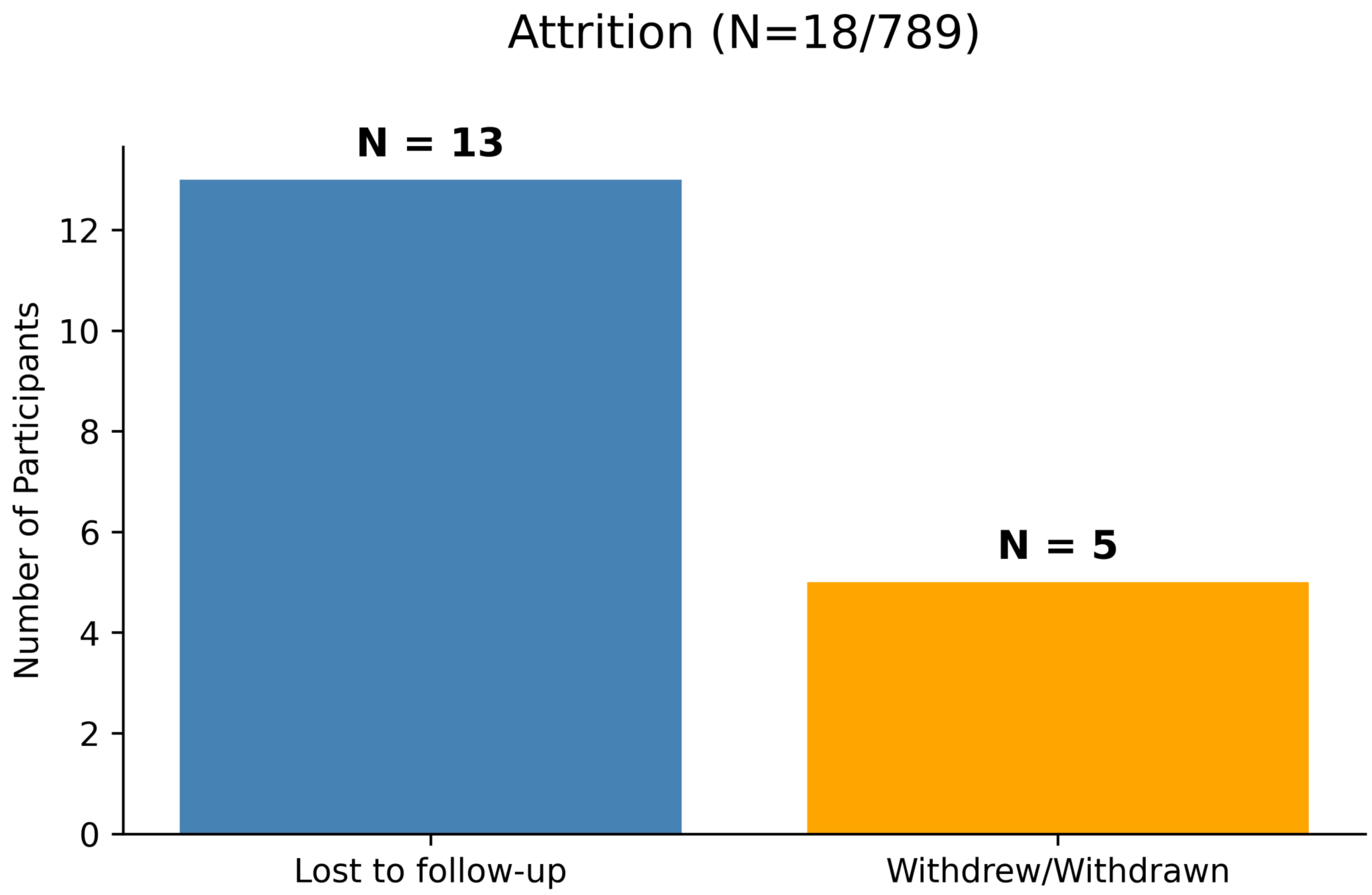

Supplemental Figure 1: Study Attrition among CARE participants. The bar chart illustrates the number of participants lost during follow-up (N = 13) and those who withdrew or were withdrawn from the study (N = 5). Counts are displayed above each bar. Attrition occurred after enrollment and before analysis. Attrition rate was 2%

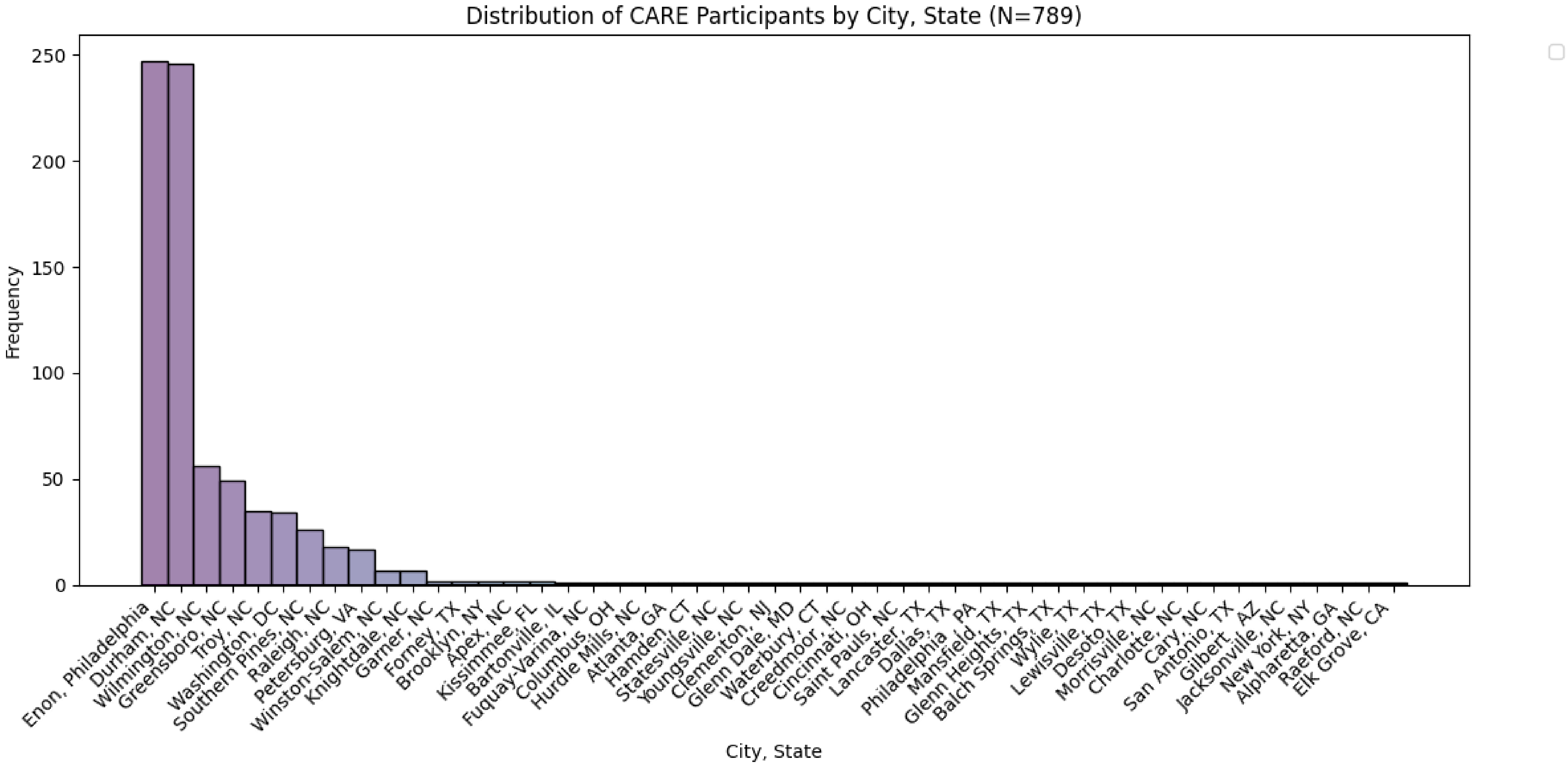

Supplemental Figure 2: Distribution of CARE Participants by City and State (N=789). Bar chart displays the geographic distribution of participants enrolled in the CARE study across various U.S. cities and states. The x-axis lists the cities and states, while the y-axis indicates the number of participants. Enon, Philadelphia had the highest participant count, followed by cities in North Carolina such as Winston-Salem, Greensboro, and the Raleigh-Durham-Chapel Hill area, as well as other locations including Washington, DC; Petersburg, VA; and Baltimore, MD.

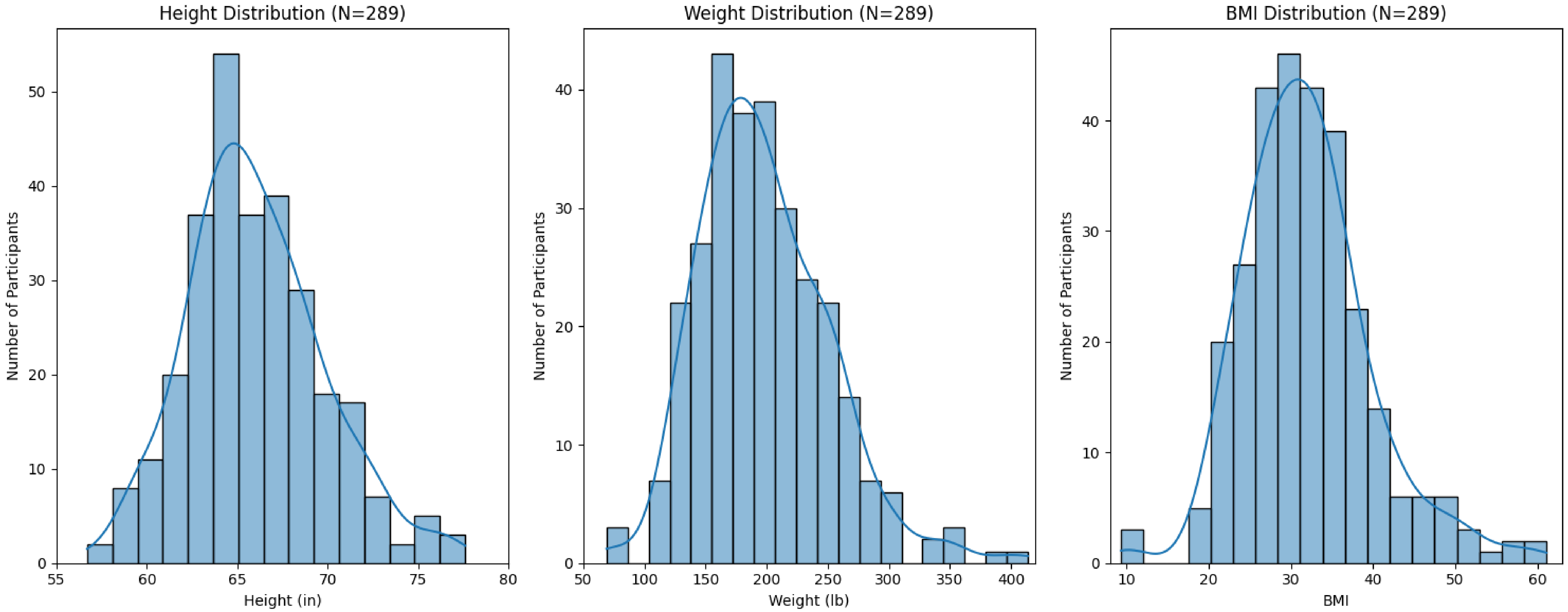

Supplemental Figure 3: Height in inches, Weight in pounds, and BMI distribution of 289 participants who were willing to have both their height and weight measured

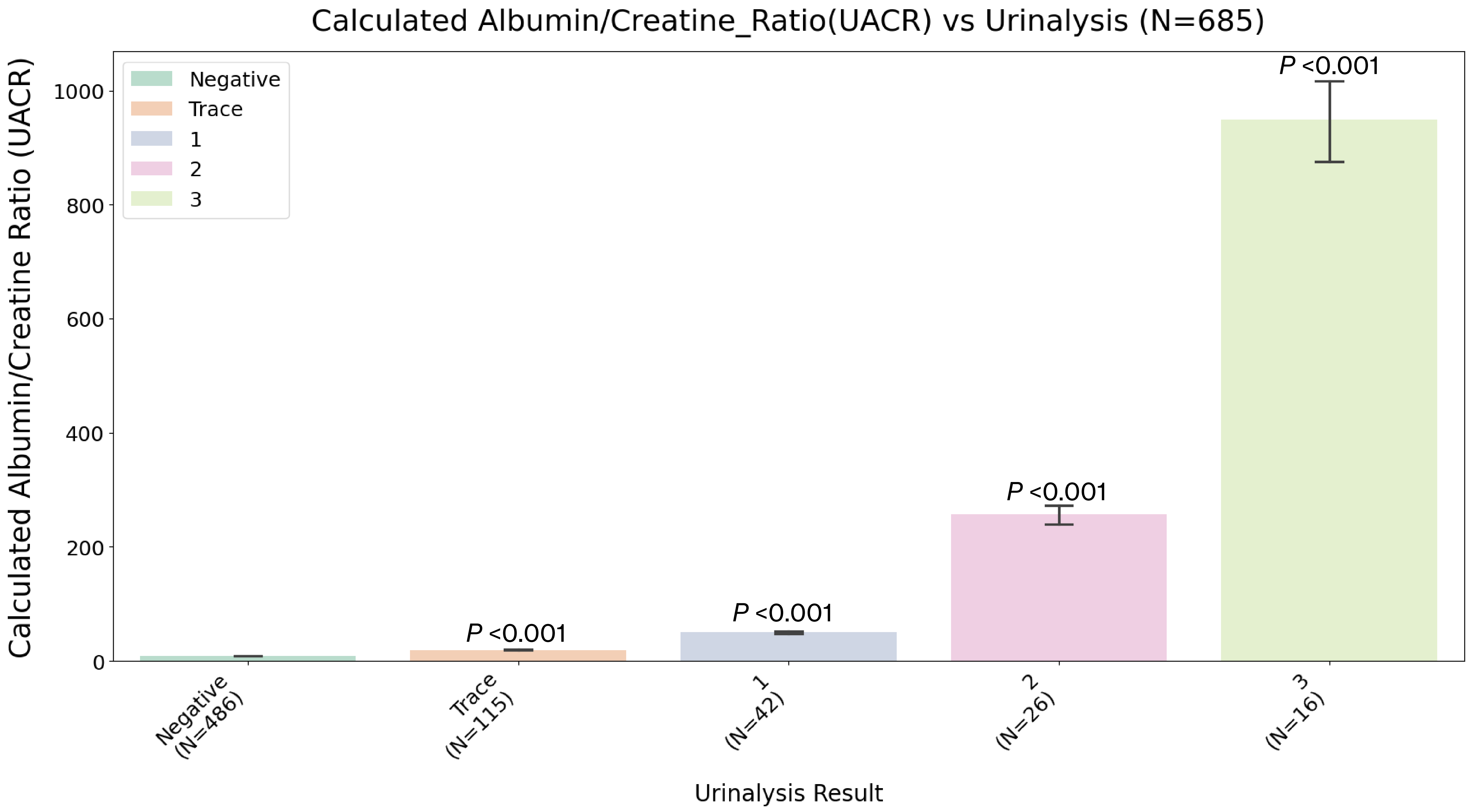

Supplemental Figure 4: Calculated Albumin/creatinine Ratio (UACR, mg/g) vs Dipstick Urinalysis. UACR T-test (Negative urinalysis vs other urinalysis results p <0.001).

Outcome	Comparison (Group 1 vs Group 2)	Effect	95% CI	Odds ratio (OR)	p	p_adj (Holm)
**Age (years) – Welch t-tests**						
	Community vs EHR	Mean diff: 7.1 years	[2.34, 11.86]		0.00429	0.0214
	Community vs Physician	Mean diff: 2.6 years	[-1.11, 6.31]		0.165	0.377
	Community vs Self	Mean diff: 9.2 years	[4.87, 13.53]		8.69×10^{-5}	5.21×10^{-4}
	EHR vs Physician	Mean diff: -4.5 years	[-10.28, 1.28]		0.126	0.377
	EHR vs Self	Mean diff: 2.1 years	[-4.08, 8.28]		0.501	0.501
	Physician vs Self	Mean diff: 6.6 years	[1.16, 12.04]		0.0180	0.0721
***APOL1* high-risk genotype – Fisher exact tests**						
	Community vs EHR	Risk diff: -0.334		0.164	1.71×10^{-7}	8.53×10^{-7}
	Community vs Physician	Risk diff: -0.461		0.0984	9.17×10^{-12}	5.50×10^{-11}
	Community vs Self	Risk diff: -0.213		0.273	3.08×10^{-4}	0.00123
	EHR vs Physician	Risk diff: -0.127		0.600	0.286	0.564
	EHR vs Self	Risk diff: +0.121		1.67	0.282	0.564
	Physician vs Self	Risk diff: +0.248		2.78	0.0317	0.0950
**Proteinuria ≥2+ (dipstick) – Fisher exact tests**						
	Community vs EHR	Risk diff: -0.674		0.0132	2.68×10^{-30}	1.61×10^{-29}
	Community vs Physician	Risk diff: -0.295		0.0650	7.08×10^{-10}	3.54×10^{-9}
	Community vs Self	Risk diff: -0.0584		0.322	0.0609	0.0609
	EHR vs Physician	Risk diff: +0.379		4.94	5.65×10^{-4}	0.00169
	EHR vs Self	Risk diff: +0.616		24.4	1.31×10^{-9}	5.22×10^{-9}
	Physician vs Self	Risk diff: +0.237		4.95	0.00786	0.0157
**Albuminuria >300 mg/g – Fisher exact tests**						
	Community vs EHR	Risk diff: -0.603		0.00678	8.22×10^{-31}	4.93×10^{-30}
	Community vs Physician	Risk diff: -0.222		0.0355	3.74×10^{-9}	1.67×10^{-8}
	Community vs Self	Risk diff: -0.0338		0.232	0.108	0.108
	EHR vs Physician	Risk diff: +0.381		5.24	4.71×10^{-4}	0.00141
	EHR vs Self	Risk diff: +0.569		34.1	3.33×10^{-9}	1.67×10^{-8}
	Physician vs Self	Risk diff: +0.188		6.52	0.0128	0.0257

Supplemental Table 4: Pairwise post-hoc comparisons among recruitment methods for age, *APOL1* high-risk genotype, proteinuria ≥2+ by urine dipstick, and albuminuria >300 mg/g. For age, pairwise Welch t-tests were performed using group means, SDs, and sample sizes, with effect estimates presented as mean difference (Group 1 − Group 2) and 95% confidence intervals. For categorical outcomes, two-sided Fisher's exact tests were used, with effect estimates presented as risk difference (RD = risk in Group 1 − risk in Group 2) and odds ratio (OR; OR>1 indicates higher odds in Group 1). Multiplicity was controlled using the Holm method within each outcome (6 pairwise comparisons per outcome); Holm-adjusted p-values are reported. Counts for categorical outcomes were derived from percentages as *round(% × N)*.

Measurements	N missing	% missing
APOL1 genotype	25	3%
Age	0	0.0%
BMI	500	63%
Dipstick protein category	104	13%
Measured UACR	682	86%
Calculated UACR	104	13%
Recruitment strategy	0	0%
Sex Assigned at birth	0	0%
eGFR (for registry eligibility)	727	92%

Supplemental Table 5: Missingness Summary (N=789). Missingness was highest for eGFR (for registry eligibility) (727 missing; 92.1%) and Measured UACR (682 missing; 86.4%), followed by BMI (500 missing; 63.4%) and both Calculated UACR and Dipstick protein category (104 missing; 13.2%). Missingness for *APOL1* genotype was low (25 missing; 3.2%), and no missing data were observed for Age, Sex Assigned at birth, or Recruitment strategy.
